# Limettin and PD98059 Mitigated Alzheimer’s Disease Like Pathology Induced by Streptozotocin in Mouse Model: Role of *p*-ERK1/2/*p-*GSK-3β/*p-*CREB/BDNF Pathway

**DOI:** 10.1007/s11481-025-10211-8

**Published:** 2025-05-17

**Authors:** Rofida M. Hassan, Nesrine S. Elsayed, Naglaa Assaf, Barbara Budzyńska, Krystyna Skalicka-Wożniak, Sherehan M. Ibrahim

**Affiliations:** 1 Department of Pharmacology and Toxicology, College of Pharmaceutical Sciences and Drug Manufacturing, Misr University for Science and Technology (MUST), 6th of October city, Giza, 12563 Egypt; 2 Department of Pharmacology and Toxicology, Faculty of Pharmacy, Cairo University, Cairo, 11562 Egypt; 3 Independent Laboratory of Behavioral Studies, Medical University of Lublin, Lublin, 20-093 Poland; 4 Department of Chemistry of Natural Products, Medical University of Lublin, Lublin, 20-093 Poland; 5 Department of Pharmacology and Toxicology, Faculty of Pharmacy, Modern University for Technology and Information (MTI), Cairo, 11571 Egypt

**Keywords:** *p-*ERK1/2, Limettin, Neuroinflammation, PD98059, Sporadic alzheimer’s disease, STZ

## Abstract

Sporadic Alzheimer’s disease (SAD) represents one of the major memory deficits that is characterized by tau hyperphosphorylation and amyloid beta (Aβ) deposition in the brain. Both are considered AD hallmarks which are mediated through neuroinflammation, oxidative stress, and cholinergic circuit interruption. This study aimed to show how limettin and PD98059 exert a neuroprotective effect against SAD and the possible role of the extracellular regulated kinase (*p-*ERK1/2) and glycogen synthase kinase-3 beta (*p-*GSK-3β) (Ser9)/cAMP-response element binding protein (*p-*CREB) (Ser133)/brain derived neurotrophic factor (BDNF) pathway. Control animals (Group I) received the vehicles, group II received PD98059 (10 mg/kg/i.p), while group III was administered limettin (15 mg/kg/i.p). Additionally, the other three groups received a single dose of streptozotocin (STZ; 3 mg/kg/ICV), where group IV served as the SAD group, while groups V and VI received PD98059 and limettin daily for 3 weeks, respectively. The SAD animals receiving PD98059 and limettin increased the number of arm entries, % alternations in Y-maze, with reduction in mean escape latency, increase in time spent in target quadrant and platform crossing in Morris Water Maze, compared to the SAD group. Additionally, PD98059 and limettin administration to the STZ group downregulated persistent activation of *p*-ERK1/2 which in turn increased *p*-GSK-3β (Ser9), leading to enhanced *p-*CREB (Ser133) and BDNF expressions, as well as reducing inflammatory markers *viz*., nuclear factor-kappa B and interleukin-6, leading to decreased Aβ deposition. Both treatments reduced immunohistochemical *p*-tau expression, brain edema, and increased intact neuron cells remarkably. Thus, based on these findings, PD98059 and limettin may have promising effects in protecting against SAD. Using blockers/inhibitory molecules are recommended to confirm effect through the corresponding pathway.

**Supplementary Information:**

The online version contains supplementary material available at 10.1007/s11481-025-10211-8.

## Introduction

The most prevalent kind of dementia, primarily affecting the elderly, is Alzheimer’s disease (AD) with preferential index to female gender (Zhang et al. 2021). The US population estimate for 2022 shows that the prevalence of sporadic Alzheimer’s disease (SAD) is 5.0% among those 65 to 74 years old, 13.1% among those 75 to 84 years old, and 33.2% among those 85 years of age and older (Lane et al. 2022).

People diagnosed with AD displayed cognitive impairment symptoms such as forgetting things, having problems in following conversations, finishing tasks or making decisions as well (Dillon et al. 2013). Furthermore, neuropsychiatric symptoms, such as depression and apathy, were observed and associated to memory decline in mild/early AD, besides verbal and physical agitation (Lyketsos et al. 2011).

It is well known that AD is characterized by neurodegeneration in hippocampal, cortical regions, and occurrence of a cholinergic imbalance stemming from oxidative stress and inflammation in neurons, resulting in excessive tau phosphorylation and aggregation of amyloid beta (Aβ) in the brain (Kumar et al. 2015). This could be due to reducing α-secretase activity while increasing β-site amyloid precursor protein cleaving enzyme (BACE-1) activity, leading to toxic Aβ accumulation in addition to neurofibrillary tangles formation (Nistor et al. 2007).

Furthermore, neuroinflammation plays role in AD pathology via the generation of various inflammatory markers such as nuclear factor-kappa B (NF-κB) which activates interleukin-6 (IL-6) that promotes further release of different pro-inflammatory cytokines resulting in the synthesis of inflammatory mediators in an endless cycle (Wang et al. 2003). As well, NF-κB activation is linked to the activation of BACE-1 followed by Aβ deposition (Tamagno et al. 2012).

Noteworthy, amyloidgenesis is linked to insulin signaling pathway as Aβ deposition accompanied by disrupting expression of some mediators, resulting in insulin receptor desensitization (Folch et al. 2018). Likewise, it was reported that insulin receptors are downregulated in AD patients to point the function of insulin signaling in memory deterioration (Burillo et al. 2021), this relation was approved via enhancing the release of acetylcholine in brain through intracerebroventricular (ICV) administration of insulin (Agrawal et al. 2008).

Moreover, glycogen synthase kinase-3 beta (GSK-3β) has been considered to play an important role in the pathogenesis of type 2 Diabetes Mellitus and AD, where it acts as “tau-kinase I” that contribute to the phosphorylation of tau protein in AD (Hoppe et al. 2010). Phosphorylation of GSK-3β at Ser9 inhibits its kinase activity (Fang et al. 2000; Giese 2009) thus enhancing long term potentiation (LTP) which represents a form of flexibility and adaptation of synapse that underlies memory formation (Tang et al. 2014). On the other hand, active *p*-GSK-3β (Tyr216) co-localizes with neurofibrillary tangles and enhance generation of the harmful Aβ 42/40 in AD (Amaral et al. 2021).

Remarkably, GSK-3β when exposed to Aβ, may suppress the phosphorylation of the cAMP-response element binding protein (CREB) more strongly, thus brain derived neurotrophic factor (BDNF) down regulation. Such molecule acts as an encouraging modifier of several types of neural plasticity in the nervous system of adults (Tang et al. 2014).

Currently in this work, ICV injection of streptozotocin (STZ), a natural compound isolated from *Streptomyces achromogenes*, in sub-diabetogenic doses of STZ (1–3 mg/kg) (Grieb 2016) has been activated microglial cells, which released large quantities of free radicals and cytokines that cause harm to neurons. (Chen et al. 2020). Besides, in accordance to previous literature, tau hyperphosphorylation and Aβ generation, as well as GSK-α and β activation, are related to STZ-induced insulin receptor desensitization. (Rajasekar et al. 2014). Likewise, Animals treated with ICV-STZ also showed a loss in their ability to learn and remember things, suggesting cholinergic dysfunction brought on by low acetylcholine levels. (Saria et al. 2015). Consequentially, because STZ model displays numerous behavioral, neurochemical, and structural alterations that are similar to those seen in human SAD, it is regarded as a widely recognized representative model of SAD (Kamat 2015; Mullins et al. 2017).

The current mainstays in Alzheimer’s therapy are donepezil and galantamine, acetylcholinesterase (AchE) inhibitors, that improve cognitive and behavioral symptoms in mild to moderate AD (Zec and Burkett 2008). Additionally, memantine, an N-methyl-D-aspartate receptor antagonist, had been accepted for management of more advanced and severe cases of the disease (Olivares et al. 2013).

Unfortunately, several side effects are observed such as nausea, vomiting, loss of appetite, muscle cramps, and diarrhea through using AchE inhibitors. Besides, dizziness, constipation, headache, and shortness of breath were reported with memantine administration. Additionally, there are serious side effects seen with anti-amyloid drugs such as aducanumab, an anti-amyloid antibody, that is taken intravenously every month, like headache, falls, and amyloid-related imaging abnormalities which is a temporary swelling with or without spots of bleeding in areas of the brain specially in ApoE ε4 gene carriers (Kataria 2021; Honig et al. 2023)Therefore, efforts have been done to nominate new treatments targeting different pathways with few or no side effects for enhancing fulfillment and quality patient’s life compliance.

PD98059, a mitogen activated protein kinase MAPK (MEK) specific inhibitor, shows promising effect against neurodegeneration through preventing the phosphorylation of tau triggered by Aβ (Rapoport and Ferreira 2000). PD98059 has been also found to have many beneficial effects in certain diseases such as neuropathy as it potentiates morphine analgesia by inhibiting levels of MAPK, NF-κB, IL-6, and enhancing anti-nociceptive IL-10 factor (Rojewska et al. 2015). Moreover, PD98059 also shields from neuronal cell destruction caused by oxidative damage in oligodendroglia cell line (Subramaniam and Unsicker 2010).

Limettin (5,7-dimethoxycoumarin), citropten, is found in bergamot oil and it is a derivative of coumarins which are abundant metabolites present in extracts from many plant families, including the Rutaceae and Asteraceae (Epifano et al. 2009). It displayed anti-inflammatory and antioxidant (Kostova et al. 2012; Seong et al. 2019; Kowalczyk et al. 2022a; Lee et al. 2022a) via hindering nitrite, prostaglandin E2, IL-6, and tumor necrosis factor– alpha in addition to upregulation of expression of the cytoprotective heme oxygenase-1 enzyme (Yu et al. 2005; Pan et al. 2010).

Likewise, aromatic ring of coumarins can attach to AchE, thereby blocking the development of Aβ-AchE conjugates as well as possessing BACE-1 inhibiting activity in comply with an in in vitro study that discussed the potential of coumarins to combat against AD focusing on the structure-activity analysis (Ali et al. 2016), so it may show promising effect against AD. This work aimed to assess the effect of limettin, as a type of furanocoumarin in amelioration of SAD and its role in modulation of *p-*ERK1/2/*p-*GSK-3β/*p-*CREB/BDNF pathway and associated neuroinflammation using PD98059.

## Materials and Methods

### Ethical Statement

Methods and procedures were followed throughout this work using US National Institutes of Health guidelines for the treatment and care of laboratory animals (NIH publication No. 85 − 23, revised 2011). The Research Ethics Committee had approved these procedures for use in experimental studies at the Faculty of Pharmacy, Cairo University, Cairo, Egypt, under number of permissions PT-(3020).

### Animals

Three to four months old, adult male mice weighing 25 to 30 g were acquired from the Faculty of Veterinary Medicine Cairo University (Giza, Egypt). They have been adapted to normal housing circumstances with a 12-hour cycle of light and dark at room temperature 24–26 °C and 60% relative humidity. Food and water were provided on a free-for-all basis.

### Chemicals

STZ, limettin, and PD98059, the ERK1/2 inhibitor, had been obtained from Sigma–Aldrich (Missouri, USA). STZ was dissolved in saline, while limettin, and PD98059 were dissolved in 1% dimethyl sulfoxide (DMSO). Further analytical-grade compounds had been bought from reputable commercial vendors. Pilot study was established for limettin dose selection (Supplementary material)(Lee et al. 2022a), while other doses were used according to the previous literatures (Di Paola et al. 2010; Grieb 2016).

### SAD Induction

Thiopental (30 mg/kg/i.p) was utilized to anesthetize the mice(Gargiulo et al. 2012), Then, STZ was injected at a dose of 3 mg/kg/ICV (Grieb 2016) using the free hand technique. The bregma could be identified by visualizing an equilateral triangle between the eyes and the center of the skull, then, the needle was inserted directly through the skin and skull into the lateral ventricle, 1 mm lateral to bregma after using downward pressure above the ears (Fronza et al. 2019; Sirwi et al. 2021).

### Experiment Design

**Fig. 1:**
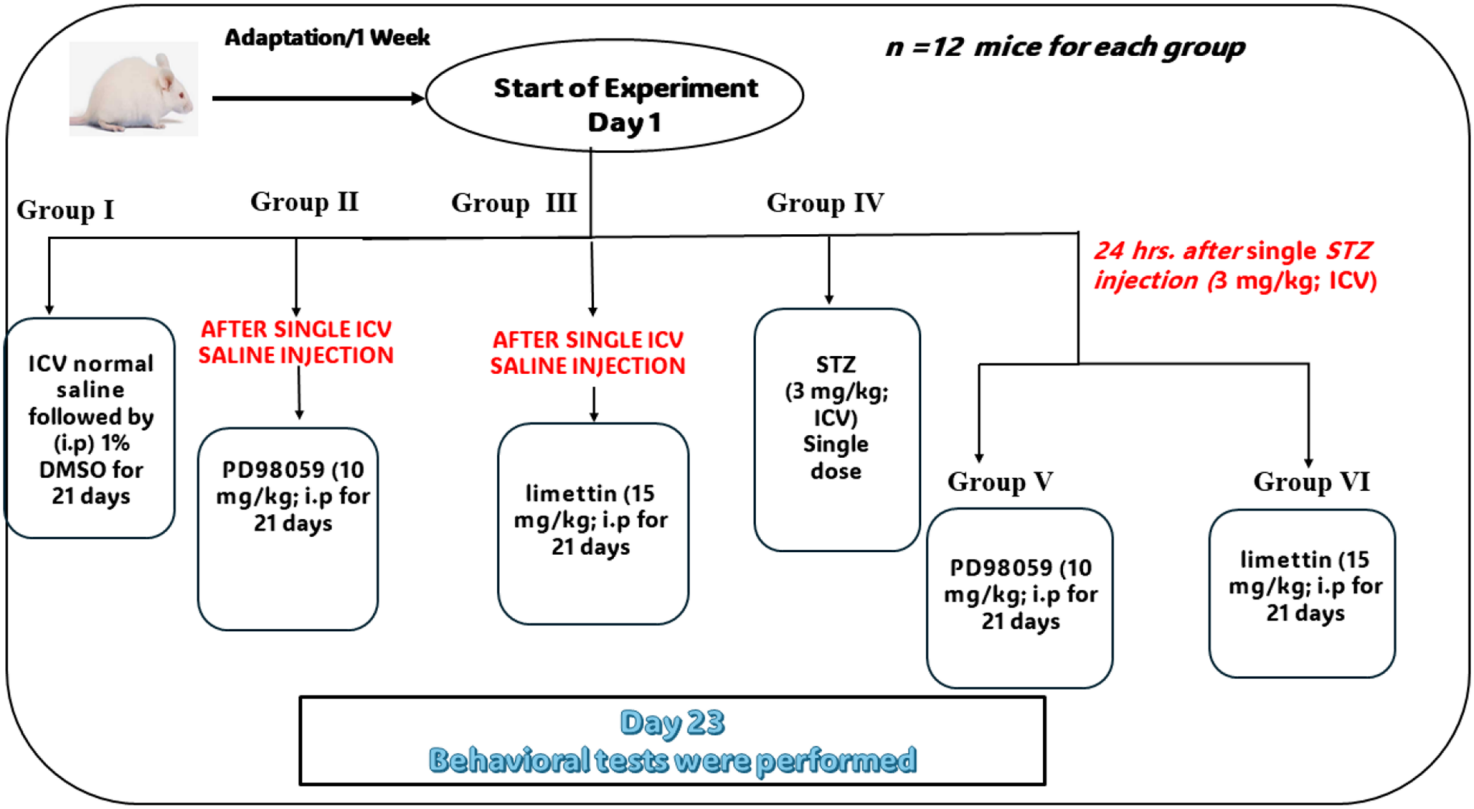
Experimental design

Animals have been randomized, divided into 6 groups and every group contained 12 mice (6 in each cage) as follows: Group I (Control group) injected normal saline via ICV route followed by intraperitoneal (i.p) 1% DMSO injection for 3 weeks and considered as the control group, while animals in groups II (PD98059 group) and III (limettin group) received PD98059 (10 mg/kg; i.p) (Di Paola et al. 2010) and limettin (15 mg/kg; i.p), respectively after single ICV saline injection and continued for 3 weeks. Moreover, mice in group IV (STZ group) were administered ICV- STZ (3 mg/kg) (Grieb 2016) as a single dose and serves as the model group. Furthermore, group V (STZ + PD98059 group) and VI (STZ + limettin group) received PD98059 (10 mg/kg/day; i.p) and limettin (15 mg/kg/day; i.p), respectively for 3 weeks, 24 h after single STZ injection (Rajkumar et al. 2022). The experimental design was illustrated in Fig. 1.

### Tissue Sampling

After 24 h of last dosing, Y-Maze and Morris Water Maze were accomplished, then animals were euthanized using cervical dislocation under an overdose of thiopental anesthesia. The whole brain (*n* = 3/group) has been preserved in 10% formalin for histopathology and immunohistochemical investigation. Hippocampus of the remaining animals were isolated and stored in **-**80 °C to assess Aβ, NF-kB, IL-6, BACE-1, and BDNF by enzyme-linked immunosorbent assay (ELISA) (*n* = 6/group), while relative protein expression of *p-*ERK1/2, *p-*GSK-3β (Ser9), and *p-*CREB (Ser133) were measured by Western Blot (*n* = 3/group).

### Assessed Parameters

#### Behavioural Test

##### Y- Maze Test

Y-maze examination assesses temporarymemory (short term) (Cognato et al. 2012) and the current study utilized a three-armed, Y-shaped maze made of wood. Every single arm extended from a central platform at a 120° angle with a length of 35 cm, height of 25 cm, and width of 10 cm. Rather than exploring the well-known arm of the maze, normal animals would rather explore a new one. Two days in a row were used to conduct the test (Rasheed et al. 2018). Every mouse was placed on the platform and given free movement for ten minutes to explore the maze on the first day, which was set aside for training. On test day, a record of every mouse’s arm entry sequence was kept for the 10-minute session. Following each mouse, 70% ethanol was used to clear the maze from any smell cues that would cause inaccurate assessment.

The consecutive entrance into every single arm of the three in the form of overlapping triad groups was recorded as an actual alternation. Possible alternations could be expressed as the overall arm entries-2 (Prieur and Jadavji 2019; Rattanapornsompong et al. 2019).

Possible Alternations = overall arm entries-2.

The spontaneous alternation percentage (SAP) was computed through dividing actual alternations by possible alternations then multiply the result by 100.

% Alternations= (Actual alternations)/ (Possible alternations) ×100.

##### Morris Water Maze (MWM)

The MWM evaluates a small rodent’s visual-spatial and visual-short-term memory skills (D’Hooge and De Deyn 2001). In the current investigation, a stainless-steel circular pool with a non-reflective internal surface having 120 cm circumference and 51 cm elevation—has been employed. With the assistance of 2 vertical strings that were fixed to the pool’s edge, The pool’s aqua level was only half of its height, the same as ambient temperature and was randomly split into four sections/ quadrants of equal size. Within the pool’s target quadrant, an immersed 10 cm in width and 28 cm in height platform has been positioned 1 cm under the aqua’s surface. Throughout the test, the platform stayed in its original position. To make the water opaque, non-toxic green dye was applied to render the platform undetectable.

In normal circumstances, animals gain up the skill of swimming straight toward the platform and arriving there faster. This procedure was carried out over the course of five days as each mouse was subjected to two successive trials on the first 4 days of the test, with an interval of at least 15 min between the trials. The maximum time for each trial was 120 s. If the mouse found the hidden platform within the 120 s, it was kept there for an additional 20 s and then removed. The mouse that failed to find the hidden platform during the designated time was gently guided onto the platform and kept there for 20 s (D’Hooge and De Deyn 2001; Vorhees and Williams 2006). On the fifth day, the probe-trial session, the platform was eliminated from the pool and the mice were given sixty seconds to explore it. The mean escape latency (MEL) on the training days and on the test day as well as the time spent in the target quadrant —where the hidden platform had been— and number of platform crossing were noted as markers of retrieval or memory (Nunez 2008).

#### Estimation of Biochemical Parameters

Mouse ELISA kits were acquired from My Bio Source, San Diego, CA, USA to assess hippocampal content of Aβ, NF-kB, IL-6, BACE-1, and BDNF (Cat No. MBS265825, MBS043224, MBS824703, MBS7234786, and MBS355435), respectively. Protein content was estimated using Bradford method (Marshall and Williams 1993). Aβ, NF-κB, IL-6, and BDNF were expressed as (pg/mg protein) while BACE-1 (ng/mg protein). Every step of the process was carried out as directed by the manufacturer.

#### Western Blot Analysis

Hippocampal protein expression of *p-*ERK1/2, *p-*GSK-3β (Ser9), and *p-*CREB (Ser133) were assessed. In brief, RIPA lysis buffer PL005, obtained from Bio BASIC INC. (Marhham Ontario L3R 8T4 Canada) was used for hippocampal protein extraction. The second step is sample separation on sodium dodecyl sulfate poly acrylamide gel electrophoresis (SDS-PAGE), which isolates proteins according to their molecular weight. After that, protein bands have been relocated to polyvinylidene fluoride (PVDF) membrane using BioRad Trans-Blot Turbo instrument. For one hour at ambient temperature, the membrane was soaked in tris-buffered saline containing 3% bovine serum albumin and Tween 20 buffer to prevent signal interference arising from non-specific interactions between the corresponding antibodies and the PVDF membrane. Incubation of the blotted target protein was done at four degrees Celsius all night with primary polyclonal antibody for *p-*ERK1/2 (Catalog No: 44-654G), *p-*GSK-3β (Ser9) (Catalog No: PA5-104555), and *p-*CREB (Catalog No: PA1-4619 ) obtained from Thermo Fisher Scientific Inc. (MA, USA). Incubation was done within horseradish peroxidase (HRP)-conjugated secondary antibody (Goat anti rabbit IgG- HRP- Goat mab -Novus Biologicals) solution over 60 min at room temperature in contrast to the blotted target protein. Finally, by utilizing stain-free technology and ChemiDoc TM imager, the acquired band magnitude has been normalized to β-actin and then viewed. Unspecified arbitrary units were used to define the values.

#### Histopathological Examination

Samples of mouse brain tissue were preserved for 72 h in 10% neutral buffered formalin, then processed through successive ethanol grades, cleaned in xylene, and inserted into paraplast tissue embedding media (Leica Biosystems). Longitudinal parts of brain ordered in serial sets (4µn thick) have been prepared to demonstrate hippocampal CA3 regions through Hematoxylin and Eosin staining to assess neuronal damage, brain matrix edema, and glial cell infilterates. Toluidine blue staining was applied to assess the mean intact neurons count neurodegeneration (Yong 1992). Focusing on CA3, as CA3 region is important for retrieving stored information based on certain cues such as recognizing certain features of the surrounding environment while the CA1 region plays a role in integration of received information mainly, and the subiculum of CA1 contributes to memory (Valenzuela and Morton 2014; Schlichting et al. 2014). Furthermore, the CA3 region has specific attention in recent years due to its involvement in memory processes, susceptibility to seizures and neuro-degeneration and the CA3 subsection has a higher degree of neuronal connection compared to other hippocampus sections (Cherubini and Miles 2015; El Tabaa et al. 2022; Rui et al. 2025). Also, upon our microscopic inspection, the CA3 region was clearly affected, and no observation was found on CA1 region.

#### Immunohistochemistry

A five-micron tissue segment immersed in paraffin has been prepared. The manufacturer’s procedure was followed throughout the immunohistochemistry testing. Brain segments have been kept with anti-*p*-tau antibody (1:100– thermofisher scientific, MA, USA, Cat. No. 44-742G) overnight at 4 °C then, for 20 min, with HRP as the secondary antibody (Envision kit DAKO), afterwards, hippocampus CA3 region of every specimen was randomly chosen, and 6 non-interconnected fields were imaged to determine the area % of *p*-tau (Abbas et al. 2021). The pyramidal neurons layer of hippocampus CA3 zone was used for immunohistochemical quantification.

### Statistical Analysis

Analysis of data has been conducted using GraphPad Prism (VER 10.4.1) program, results were represented as mean ± SD through one-way analysis of variance (ANOVA) followed by Tukey’s multiple comparison test. Level of significance was applied at *p* < 0.05. However, a two-way ANOVA with Tukey’s multiple comparisons tests were utilized to analyze the learning performance for 4 days in the MWM trial in mean escape latency (MEL). Moreover, to compare the histopathological scoring, the Kruskal-Walli’s test followed by Dunn’s post hoc test for multiple comparison was used.

## Results

The findings showed that there was no significant difference between control, PD98059, and limettin groups, therefore the model and treated groups were compared to the control group.

### Effect of PD98059 and Limettin on Y-Maze Overall Arm Entries and SAP in SAD Mice Model

**Fig. 2:**
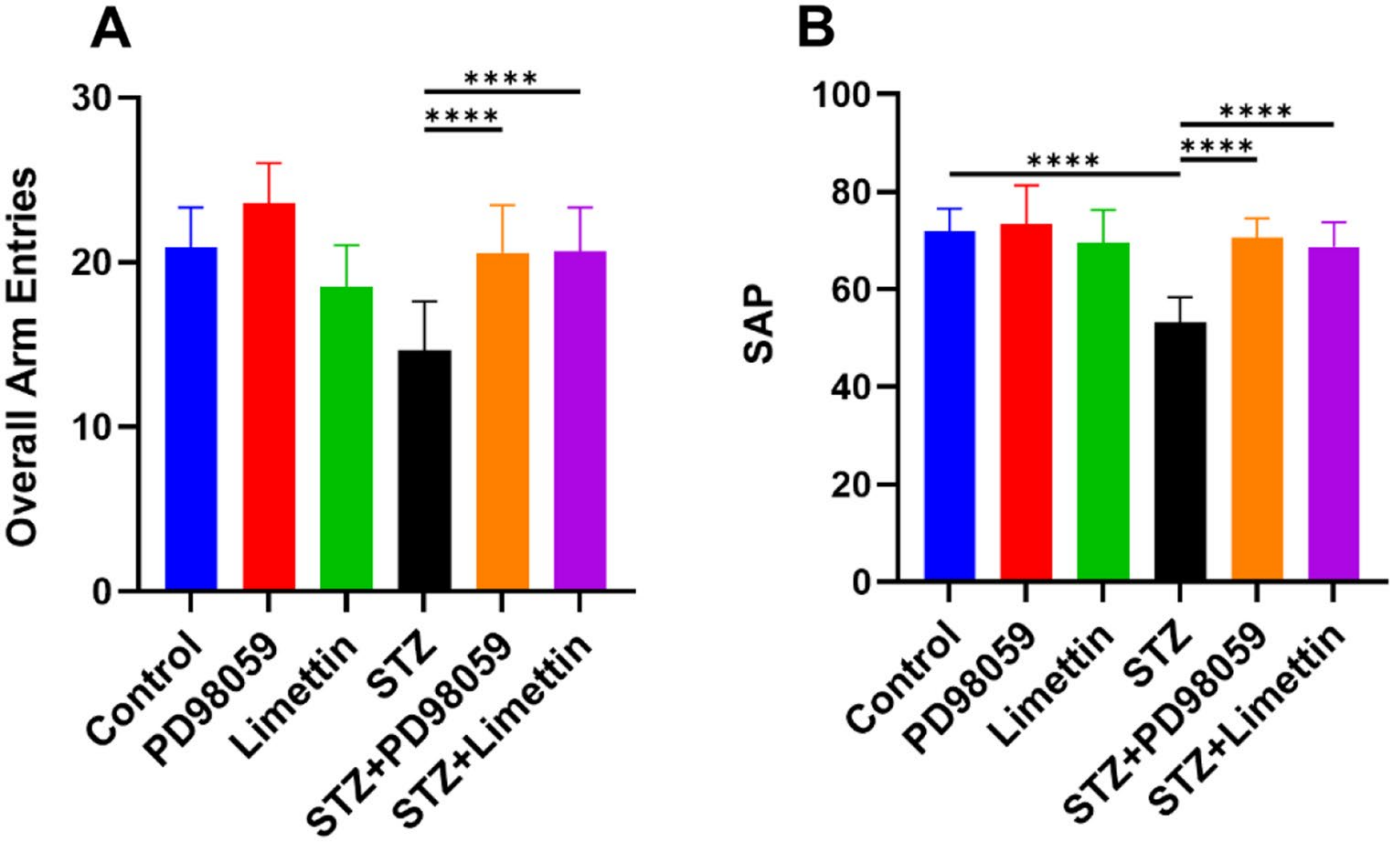
Effect of PD98059 and limettin on Y-maze overall arm entries and SAP in SAD mice model. PD98059 (10 mg/kg; i.p) and limettin (15 mg/kg; i.p) had been provided during a 21-day period post single ICV-STZ injection-induced SAD in mice. Data is set as mean ± SD; (*n* = 12), the asterisks (****) is statically significant at *p* < 0.0001, tested through Tukey’s multiple comparisons following One Way ANOVA. ANOVA: Analysis of variance, SAD: Sporadic Alzheimer disease, SAP: Spontaneous alteration percentage; STZ: Streptozotocin

The STZ injected mice exhibited lowering in the overall number of entries into each arm (Fig. 2A) as well as SAP (Fig. 2B) to 70%(14.62 ± 2.93; *p* < 0.0001) and 74% (53.19 ± 5.17; *p* < 0.0001), respectively, in comparison with control animals, while in the STZ + PD98059 and STZ + limettin groups, the overall number of entries into each arm is increased by 40% for both (20.58 ± 2.88; *p* < 0.0001; STZ + PD98059) (20.67 ± 2.67; *p* < 0.0001; STZ + limettin), while SAP elevated by 33% (70.54 ± 3.97; *p* < 0.0001) and 30% (68.68 ± 4.99; *p* < 0.0001), respectively, as compared to STZ model mice. Thus, PD98059 and limettin lessened the short-term memory impairment caused by STZ.

### Effect of PD98059 and Limettin on MWM Mean Escape Latency (MEL), the time Spent in the Target Quadrant, and Platform Crossing in SAD Mice Model

**Fig. 3:**
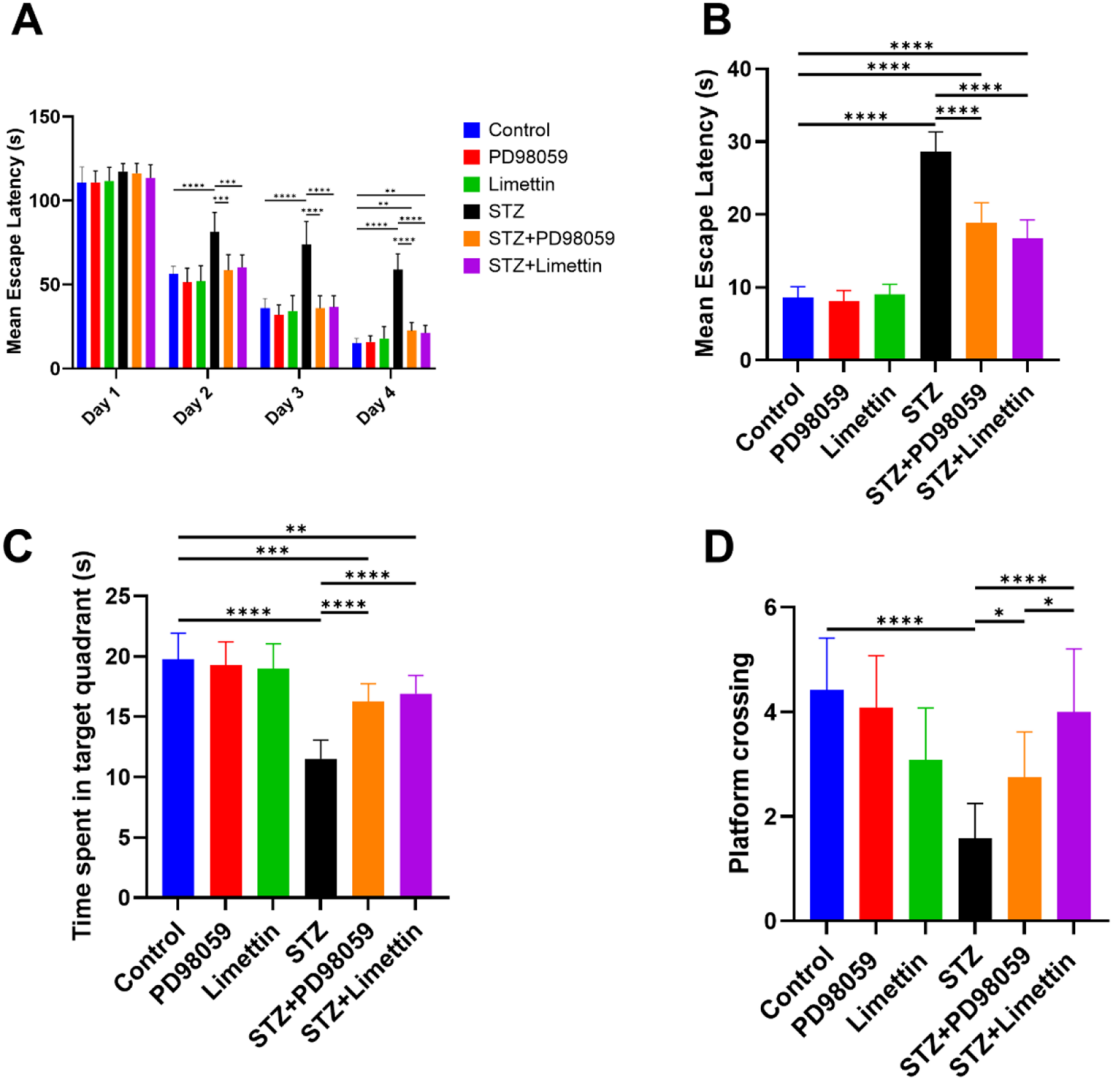
Effect of PD98059 and limettin on MWM (**A**) Mean escape latency (MEL) on trial days, (**B**) MEL on the test day, (**C**) time spent in the target quadrant, and (**D**) platform crossing in SAD mice model. PD98059 (10 mg/kg; i.p) and limettin (15 mg/kg; i.p) had been provided during a 21-day period post single ICV-STZ injection-induced SAD in mice. Data is set as mean ± SD; (*n* = 12), the asterisks (****) show statical significance at *p* < 0.0001, (***) at *p* < 0.001, (**) at *p* < 0.01 and (*) at *p* < 0.05, tested through Tukey’s multiple comparisons following One Way ANOVA. However, a two-way ANOVA with Tukey’s multiple comparisons test was utilized to analyze MEL in training days. ANOVA: Analysis of variance, MEL: Mean escape latency, MWM: Morris water maze, SAD: Sporadic Alzheimer’s disease, STZ: Streptozotocin

On training days, MEL was measured and all groups showed no significant difference on the first day, while there was significant improvement in learning and memory on 2^nd^, 3^rd^, 4^th^ day in PD98059 and limettin treated mice, compared to STZ group (Fig. 3A). On the test day, the model group increased the MEL (Fig. 3B) by 234% (28.67 ± 2.67; *p* < 0.0001), in comparison with control group. Notably, PD98059 and limettin treated mice displayed significant MEL reduction compared to STZ model group by 35% (18.83 ± 2.79; *p* < 0.0001) and 42% (16.75 ± 2.53; *p* < 0.0001), respectively. Moreover, STZ model group revealed significant decline in the time spent in the target quadrant (Fig. 3C) in comparison with control animals by 41% (11.50 ± 1.57; *p* < 0.0001), while PD98059 and limettin treated groups showed marked rise in the time spent in the target quadrant, in comparison with STZ model group by 41% (16.25 ± 1.49; *p* < 0.0001) and 48% (16.92 ± 1.51; *p* < 0.0001) respectively. STZ also decreased the number of platform crossing (Fig. 3D) significantly by 64% (1.58 ± 0.67; *p* < 0.0001), as compared to the control animals, while both PD98059 and limettin treated groups showed marked increase in platform crossing by 74% (2.750 ± 0.87; *p* < 0.05) and 153% (4.00 ± 1.21; *p* < 0.0001), respectively as compared to model group. Additionally, limettin treated group showed significant increase in platform crossing more than PD98059 by 46% (4.00 ± 1.21; *p* < 0.05).

### Effect of PD98059 and Limettin on *p-*ERK1/2, *p-*GSK-3β (Ser9), *p-*CREB(Ser133) Expression, as Well as BDNF Hippocampal Content in SAD Mice Model

**Fig. 4:**
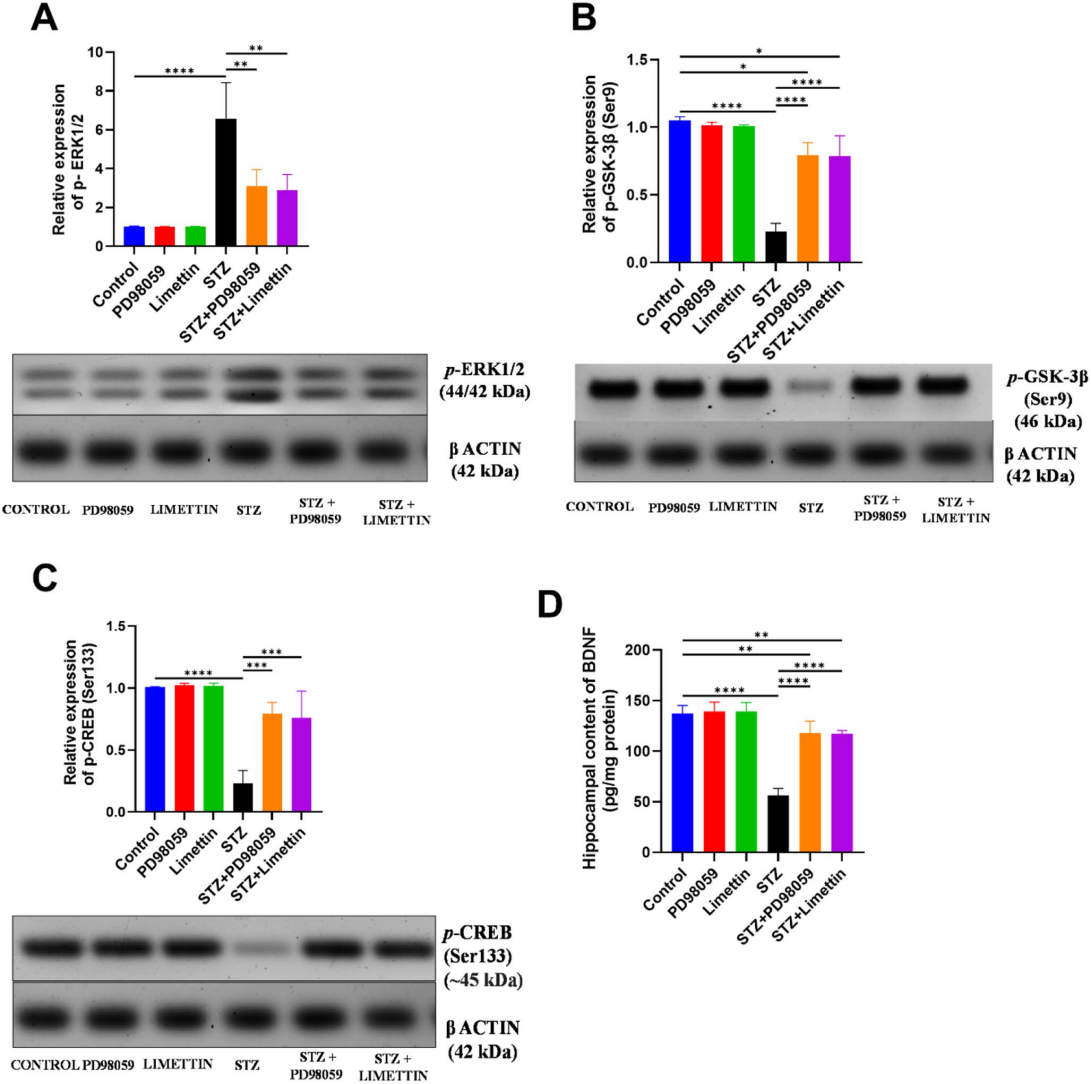
Effect of PD98059 and limettin on hippocampal content of (**A**) *p-*ERK1/2, (**B**) *p-*GSK-3β (Ser9), (**C**) *p-*CREB (Ser133) expressions, and (**D**) BDNF and their corresponding blots in SAD mice model. PD98059 (10 mg/kg; i.p) and limettin (15 mg/kg; i.p) had been provided during a 21-day period post single ICV-STZ injection-induced SAD in mice. Data is set as mean ± SD; (*n* = 3–6), the asterisks (****) show statical significance at *p* < 0.0001, (***) at *p* < 0.001, (**) at *p* < 0.01 and (*) at *p* < 0.05, tested through Tukey’s multiple comparisons following One Way ANOVA. ANOVA: Analysis of variance, BDNF: Brain derived neurotrophic factor, CREB: cAMP-response element binding protein, ERK1/2: Extracellular regulated kinase, GSK-3β: Glycogen synthase kinase-3 beta, SAD: Sporadic Alzheimer’s disease, STZ: Streptozotocin

The expression of *p*-ERK1/2 (Fig. 4A) was upregulated to 6.4-folds (6.58 ± 1.85; *p* < 0.0001) while *p*-GSK-3β (Ser9) (Fig. 4B) is downregulated to 22% (0.23 ± 0.06; *p* < 0.0001) within model animals, as compared to the control group. The STZ + PD98059 and STZ + limettin groups downregulated *p*-ERK1/2 expression to 47% (3.12 ± 0.83; *p* < 0.01) and 44% (2.9 ± 0.80; *p* < 0.01), while both treatments upregulated *p*-GSK-3β (Ser9) by 2.4 folds (0.79 ± 0.09; *p* < 0.0001; STZ + PD98059), (0.79 ± 0.15; *p* < 0.0001; STZ + limettin), as compared to the model group. Both *p-*CREB (Ser133) expression (Fig. 4C) and BDNF content (Fig. 4D) were decreased throughout the model animals to 23% (0.23 ± 0.10; *p* < 0.0001) and 41%(56.32 ± 6.91; *p* < 0.0001), respectively, compared to the healthy mice. In STZ + PD98059 group, *p-*CREB (Ser133) and BDNF increased by 240% (0.79 ± 0.09; *p* < 0.001) and 109% (117.7 ± 12.03; *p* < 0.0001), respectively, while the STZ + limettin group stimulated these molecules by 230% (0.76 ± 0.22; *p* < 0.001) and 108% (117.4 ± 2.90; *p* < 0.0001), respectively, in comparison with STZ-induced SAD mice.

### Effect of PD98059 and Limettin on STZ-induced Neuroinflammation and Aβ Deposition Related Markers in SAD Mice Model

**Fig. 5:**
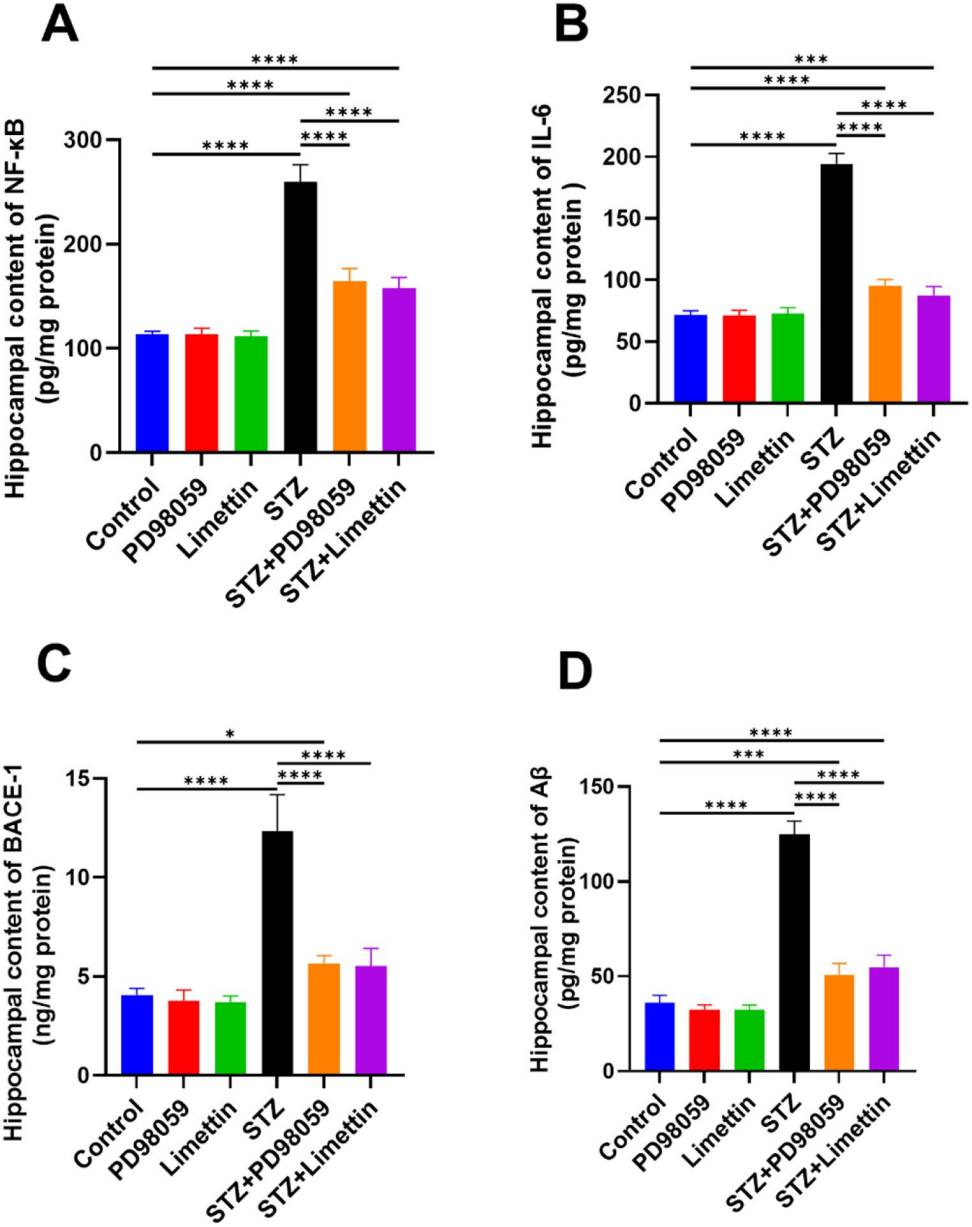
Effect of PD98059 and limettin on hippocampal content of (**A**)NF-κB, (**B**) IL-6, (**C**) BACE-1, and (**D**) Aβ in SAD mice model. PD98059 (10 mg/kg; i.p) and limettin (15 mg/kg; i.p) had been provided during a 21-day period post single ICV-STZ injection-induced SAD in mice. Data is set as mean ± SD; (*n* = 6), the asterisks (****) show statical significance at *p* < 0.0001, (***) at *p* < 0.001, and (*) at *p* < 0.05, tested through Tukey’s multiple comparisons following One Way ANOVA. Aβ: Amyloid beta, ANOVA: Analysis of variance, BACE-1; β-site amyloid precursor protein cleaving enzyme 1, IL-6: Interleukin 6, NF-κB: Nuclear factor-kappa B, SAD: Sporadic Alzheimer’s disease, STZ: Streptozotocin

The ICV-STZ group enhanced NF-κB (Fig. 5A), IL-6 (Fig. 5B), BACE-1 (Fig. 5C), and Aβ (Fig. 5D) by 129% (259.3 ± 16.85; *p* < 0.0001), 170% (194.1 ± 8.45; *p* < 0.0001), 203% (12.35 ± 1.83; *p* < 0.0001) and 247% (124.7 ± 7.19; *p* < 0.0001), respectively, as compared to the control group. The STZ + PD98059 group reduced NF-κB, IL-6, BACE-1, and Aβ by 37% (164.1 ± 12.47; *p* < 0.0001), 51% (95.56 ± 4.93; *p* < 0.0001), 52.5% (5.67 ± 0.39; *p* < 0.0001), and 59% (50.88 ± 5.98; *p* < 0.0001), respectively, while the STZ + limettin group decreased them by 39% (157.7 ± 10.39; *p* < 0.0001), 55% (87.40 ± 7.50; *p* < 0.0001), 54% (5.52 ± 0.90; *p* < 0.0001), and 56% (54.92 ± 6.25; *p* < 0.0001), respectively, as compared to the SAD group.

### Effect of PD98059& Limettin on Hippocampal *p*-tau in in SAD Mice Model

**Fig. 6:**
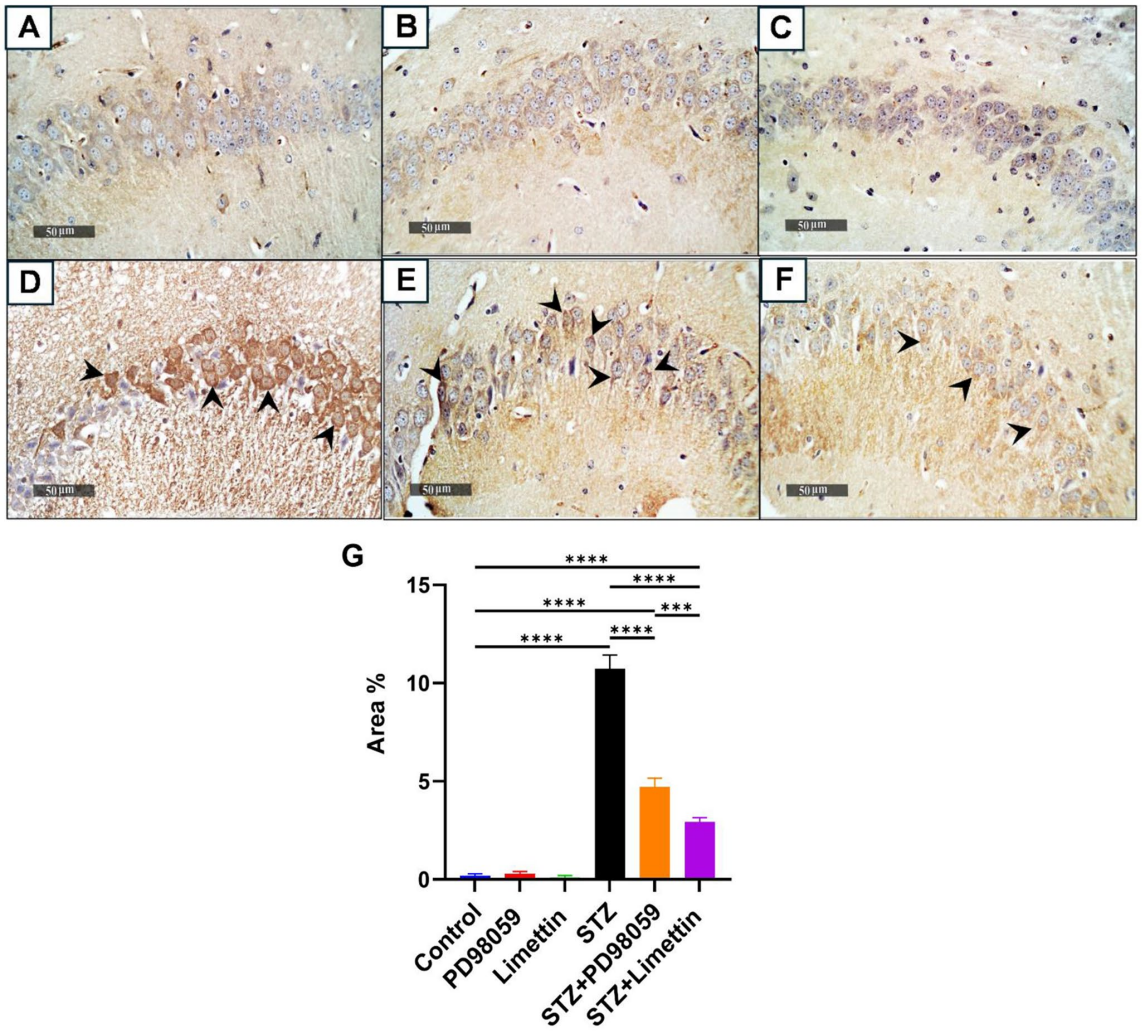
Effect of limettin and PD98059 on hippocampal expression of *p*-tau in SAD mice model. PD98059 (10 mg/kg; i.p) and limettin (15 mg/kg; i.p) had been provided during a 21-day period post single ICV-STZ injection-induced SAD in mice. Sections of (**A**) control group, (**B**) PD98059, and (**C**) limettin groups displayed null expression of phosphorylated tau in the brain tissue, while expression of phosphorylated tau in the brain tissue was high in (**D**) STZ received group showed by black arrows. In (**E**) STZ + PD98059 group showed reduction in the deposition of phosphorylated tau in brain tissue, additionally, (**F**) STZ + limettin group showed marked downregulation of phosphorylated tau in brain tissue. These results were summarized as area percentage of immunohistochemical expression levels of *p*-tau in different experimental groups in (**G**). Data is set as mean ± SD; (*n* = 3), the asterisks (****) show statical significance at *p* < 0.0001, and (***) at *p* < 0.001, tested through Tukey’s multiple comparisons following One Way ANOVA. ANOVA: Analysis of variance, *p*-tau: Phosphorylated tau, SAD: Sporadic Alzheimer’s disease, STZ: Streptozotocin

The control mice (Fig. 6A), PD98059 group (Fig. 6B), and limettin group (Fig. 6C) showed null *p*-tau expression in the brain tissue. Upregulated immunohistochemical expression of *p*-tau in the brain tissue were detected in ICV-STZ group (Fig. 6D) to 55 folds (10.73 ± 0.70; *p* < 0.0001) as compared to the control group. Administration of PD98059 in STZ-induced SAD group (Fig. 6E) resulted in a reduction in the deposition of *p*-tau in brain tissue to 43% (4.70 ± 0.46; *p* < 0.0001), as compared to the model group. Moreover, the STZ + limettin group (Fig. 6F) showed a marked reduction in the deposition of *p*-tau in brain tissue to 26% (2.93 ± 0.21; *p* < 0.0001) and 62% (2.93 ± 0.21; *p* < 0.001) as compared to the model and STZ + PD98059 group, respectively. The immunohistochemical expression was presented as area % as shown in (Fig. 6G).

### Effect of PD98059 and Limettin on Abnormalities Occurred in hippocampus Histology in SAD Mice Model

**Fig. 7:**
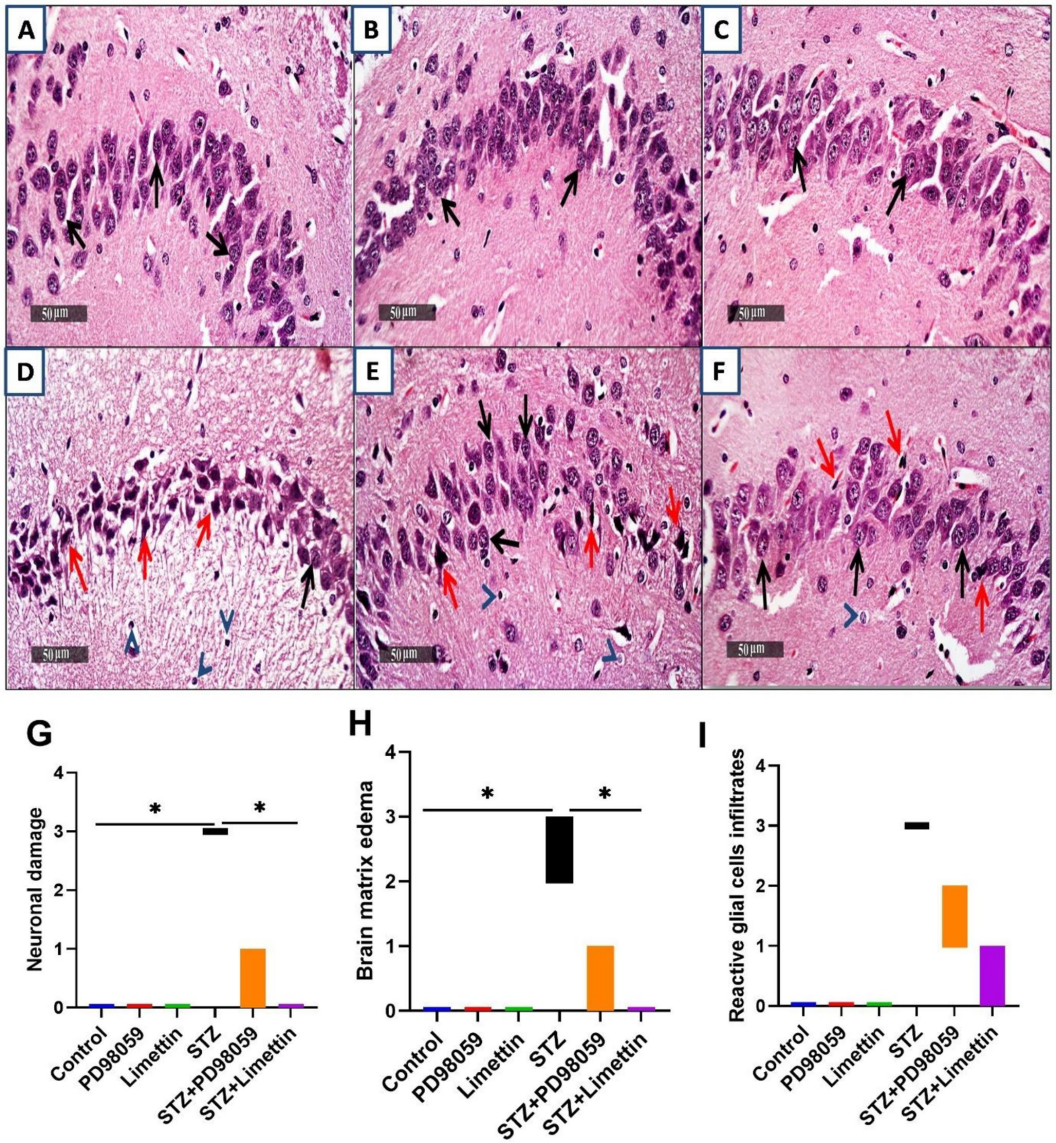
Effect of PD98059 & limettin on histopathological changes and scoring in hippocampal CA3 regions in SAD mice model using (H&E) staining (*n* = 3). PD98059 (10 mg/kg; i.p) and limettin (15 mg/kg; i.p) had been provided during a 21-day period post single ICV-STZ injection-induced SAD in mice. Sections of (**A**) control group, (**B**) PD98059-recieving group, as well as (**C**) limettin receiving group showed normal structure of different neurons in hippocampus without abnormal histological alterations. In (**D**) ICV-STZ injected group, severe neuronal loss and neuronal degenerative changes alternated with abundant figures of hyper-eosinophilic, shrunken pyramidal neurons with indistinct intracellular details (**red arrow**) having a small number of dispersed, seemingly undamaged cells (**black arrow**), sever edema with greater records of glial cells reactivity (**arrowhead**) were observed. In (**E**) STZ + PD98059 group, demonstrated mild persistent records of neuronal degenerative changes (**red arrow**) interleaved with elevated clearly visible intact neurons (**black arrow**) with coexistence of interacting penetrative glial cells (**arrow head**) and minimal edema of intercellular brain matrix in different layers, while in (**F**) STZ + limettin group, samples showed significant higher neurological protective effects altogether with minimal sporadic few records of degenerated cells (**red arrow**) and copious figures of apparent robust well-structured cells (**black arrow**), few persistent numbers of reactive glial cells infiltrates (**arrow head**) (50 μm). Histopathological scoring in (**G**) neuronal damage, (**H**) brain matrix edema and (**I**) reactive glial infiltrates were made using the Kruskal-Walli’s test followed by Dunn’s post hoc test for multiple comparison the asterisk (*) shows statistical significance at *p* < 0.05. SAD: Sporadic Alzheimer’s disease, STZ: Streptozotocin

The microscopic inspection of the CA3 areas of the hippocampus in several samples showed that the samples in the control group (Fig. 7A) had ordinary hippocampal morphology, unchanged intercellular matrix, and little reactivity of glial cells in addition to unharmed, properly organized pyramidal neural networks with intact nuclear and internal cellular structure. Similarly, the PD98059 group (Fig. 7B) and limettin group (Fig. 7C) samples exhibited identical data as the control group with no unusual histological changes. On contrary, the STZ model group (Fig. 7D) showed severe neuronal loss and neuronal degenerative changes with abundant figures of hyperesenophilic, shrunken pyramidal neurons with vague intracellular findings alternated with number of dispersed, undamaged cells, severe swelling in brain matrix and greater numbers of infiltration of reactive glial cells. Administration of PD98059 to STZ-induced SAD group (Fig. 7E) showed mild persistent degenerative alterations in neurons combined to higher records of seemingly intact brain cells with coexistence of penetrative reactive glial cells and minimal swelling of intrinsic neurological matrices in different layers. Moreover, limettin administration to STZ-induced SAD group (Fig. 7F) has shown considerably greater neuroprotective action compared to PD98059 with minimal intermittent few records of degenerated neurons and abundant numbers of apparent well-structured neurons, low records of reactive glial cells infiltration. Furthermore histopathological scoring illustrating neural morphology data was shown in (Fig. 7G) neuronal damage, (Fig. 7H) brain matrix edema, and (Fig. 7I) glial cell infiltrate to display the improvement through treatments administration.

### Effect of PD98059 and Limettin on Neuron Cells Viability in SAD Mice Model

**Fig. 8:**
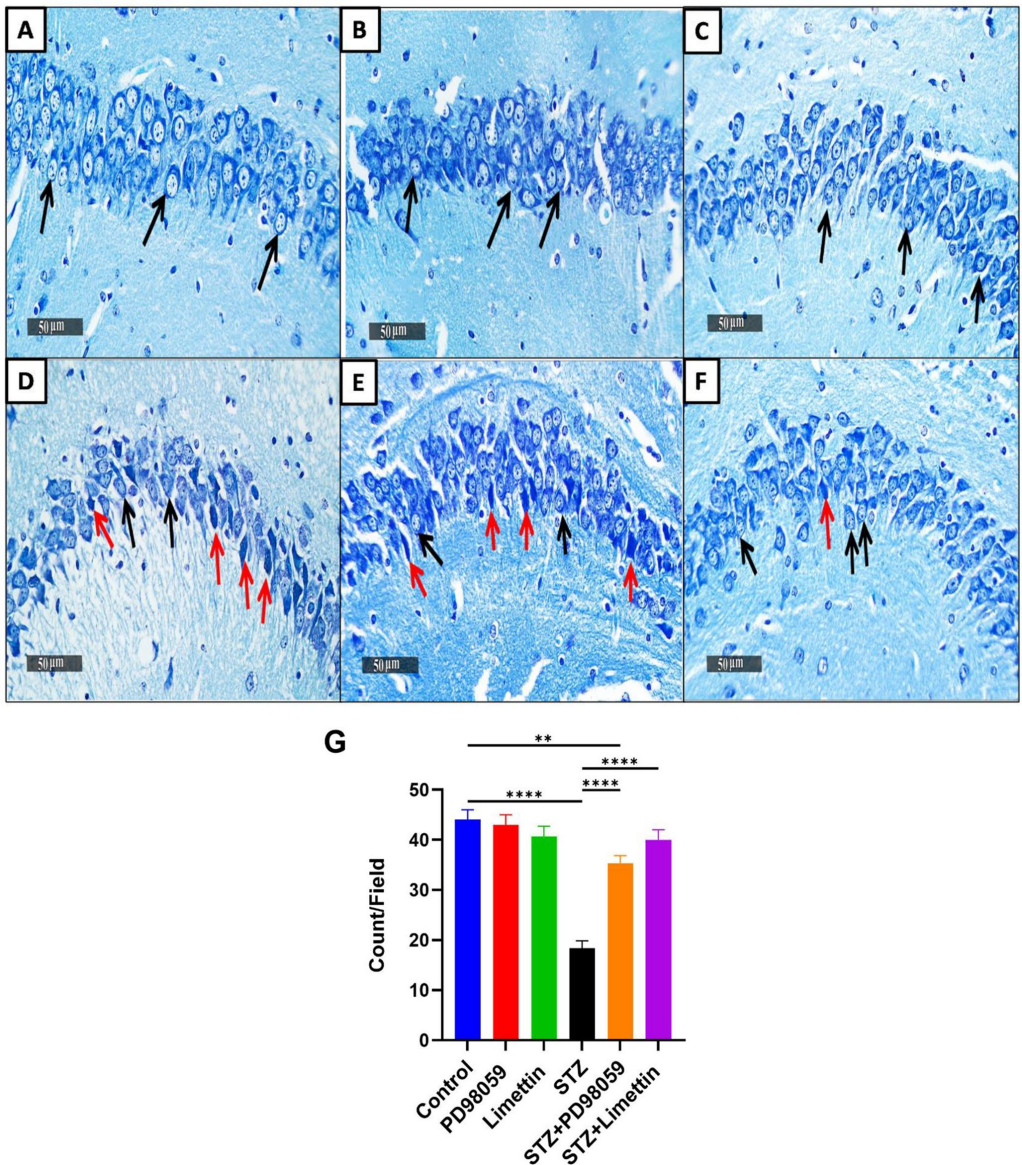
Effect of PD98059 & limettin on neuron cells using Toluidine blue stain in SAD mice model. PD98059 (10 mg/kg; i.p) and limettin (15 mg/kg; i.p) had been provided during a 21-day period post single ICV-STZ injection-induced SAD in mice. The count of intact neurons was inspected in the hippocampal CA3 regions and demonstrated variance between several groups. (**A**) Control group, (**B**) PD98059, and (**C**) limettin groups showed no significant difference in intact neuron cells. Moreover, (**D**) animals injected with STZ exhibited a significant drop in intact neuronal cells, while (**E**) STZ + PD98059 group displayed significant improvement in the intact neuron count in CA3 region. Similarly, (**F**) STZ + limettin group showed preservation of intact neurons. These results were summarized as count /field in different experimental groups in (**G**). **Black arrows** show intact neurons while **red arrows** show shrunken, dark degenerated neurons (50 μm). Data is set as mean ± SD; (*n* = 3), the asterisks (****) show statical significance at *p* < 0.0001 and (**) at *p* < 0.01, tested through Tukey’s multiple comparisons following One Way ANOVA. ANOVA: Analysis of variance, SAD: Sporadic Alzheimer’s disease, STZ: Streptozotocin

The control group (Fig. 8A) as well as animals received either PD98059 (Fig. 8B) or limettin (Fig. 8C) showed no significant difference in intact neuron cells, while the mice injected by STZ (Fig. 8D) indicated a decline in intact neuron cells to 41% (18.33 ± 1.53; *p* < 0.0001), as compared to the control group. The STZ + PD98059- group (Fig. 8E) showed significant improvement in the intact neuron count in CA3 region to 1.9-folds (35.33 ± 1.53; *p* < 0.0001), as compared to the STZ receiving mice. Additionally, the STZ + limettin group (Fig. 8F) increased intact neurons to 2.2-fold (40.00 ± 2.00; *p* < 0.0001), as compared to the model group These results were summarized as count /field in different experimental groups in (Fig. 8G).

## Discussion

Development of SAD may occur due to the complicated interplay of the environment factors, lifestyle, and genes but ageing remains the biggest risk factor for SAD. SAD is typified by the buildup of tau protein filaments and plaques containing amyloid which is directly linked to the neurodegenerative, inflammatory, and oxidative stress processes in patients’ brains (Scheltens et al. 2016). This is the first study for assessment purposes of the possible protecting impact of limettin in STZ-induced SAD in mice, beside elaborating the role of *p-*ERK1/2 and *p-*GSK-3β/ *p-*CREB/ BDNF pathway through using PD98059.

Administration of either PD98059 or limettin to STZ group improved hippocampal histopathological alterations that were deteriorated in the SAD group. Noteworthy, the treated animals displayed enhanced cognitive and memory performance in the behavioral tests reflected by improved SAP and number of arm entries in Y-maze, beside MEL, time spent in the target quadrant, and platform crossing in MWM when compared to the STZ receiving animals. Furthermore, STZ + PD98059 and STZ + limettin groups potentiated the neuroprotective arm through inhibition of the persistent activation of *p*-ERK1/2 which in turn caused upregulation of *p*-GSK-3β (Ser9), leading to the upregulation of *p*-CREB (Ser133) and BDNF. Additionally, PD98059 and limettin treated groups suppressed neuroinflammation through lowering NF-κB which in turn suppressed IL-6, thus reducing BACE-1 content, Aβ formation and downregulating *p*-tau expression, compared to the STZ group. These changes were reflected on neuronal viability observed by Toluidine blue staining.

The ICV-STZ group in the present study demonstrated a significant decline in both learning and memory processes, as evidenced by increasing MEL, a reduction in the time spent in the target quadrant, and times of platform crossing as well in the MWM in addition to reduced SAP recorded in Y-maze test, respectively. This in comply with a study documented that STZ affected mice behaviors related to cognition and memory (Zou et al. 2017). The current study showed that STZ + PD98059 and STZ + limitten groups displayed marked improvement in memory and learning functions manifested by enhancing behavioral battery to go align with the study that discussed how ERK1/2 inhibition by PD98059 enhanced MWM results by improving spatial memory in Aβ-injected rats (Ashabi et al. 2012). In addition to another study that reported ameliorating mice behaviors in lipopolysaccharide–induced memory deficit model using imperatorin, a furanocoumarin compounds that mimics limettin (Chowdhury et al. 2018).

In this work, ICV-STZ group increased the expression of *p*-ERK1/2 and downregulated *p*-GSK-3β (Ser9), beside reducing the hippocampal content of *p*-CREB (Ser133) and BDNF, thus participating in neuronal death and AD pathogenesis. These were in parallel with several studies that documented enhanced glomerular ERK1/2 phosphorylation in STZ-induced hyperglycemia in rats and STZ-induced SAD (Mage et al. 2002; Javadpour et al. 2021). Persistent upregulation in *p*-ERK1/2 may be involved in AD pathogenesis through its phosphorylation resulting in proliferation, activation of microglial cells, and secretion of cytokines which interfere with the role of BDNF as a neuroprotective and anti-inflammatory mediator. This in comply with previous in vivo study of spinal cord injury model as well as Aβ 1–42 oligomers injection in mice AD model (Morroni et al. 2016; Liang et al. 2018) and in vitro AD study utilizing Sprague Dawley rat neonatal cell cortex in cell culture (Tang et al. 2014). These results pinpoint the role of persistent stimulation of *p-*ERK1/2 with corresponding BDNF expression reduction in AD models. Likewise, another study documented reducing synaptogenesis by excessive exposure to fluoride via increasing *p*-ERK1/2, thus hippocampal BDNF downregulation in the of Sprague-Dawley rats (Chen et al. 2018). Of note, STZ + PD98059 and STZ + limettin groups reversed the previous alterations to ameliorate deterioration reported in STZ- injected mice. Furthermore, ERK1/2 phosphorylation inhibition by STZ + PD98059 group may participate in AD amelioration which may be supported by an in vitro study used hippocampal slice cultures of male Wistar rats to report that ERK1/2 activation is observed shortly following the lack of oxygen and glucose in CA1 and CA3 of hippocampus. However, the restriction of this axis by PD98059 or U0126 provided a limited level of defense towards this damage (Rundén-Pran et al. 2005). Likewise, some reported how Citrus aurantifolia oil inhibited the growth of smooth muscle cells in blood vessels through inhibition of ERK1/2 phosphorylation (Song et al. 2022) which may clarify the inhibitory effect of STZ + limittin group on ERK1/2, similarly. Another in vitro study of melanogenesis induction in melanoma cell line showed the ERK1/2 inhibition by citropten/ limettin alone or combined with U0126, an ERK1/2 inhibitor, increased phosphorylation of CREB (Alesiani et al. 2009).

Surprisingly, ERK1/2 role in AD pathogenesis is controversial as it was documented that either activation or inhibition of ERK1/2 pathway may result in SAD progress. Herein, inhibition of ERK1/2 could halt SAD features, while some investigations revealed that ERK1/2 has positive effect on neuroplasticity, IL-10 regulation (Correa et al. 2010), hence maintaining ERK1/2 activation guards versus neuroinflammation and damage caused by Aβ (Ishii et al. 2016; De Araújo et al. 2022). This conflict can be resolved based on the finding that discussed how over- or under-activation of ERK1/2 may be related to AD pathogenesis. It has been found that this depends on the illness’s stage/level and the brain region for example, it was reported that ERK1/2 is greater in trans-entorhinal extending neuronal axons in the early phases of neurodegeneration, while throughout the latter disease stages in neuronal cell bodies and dystrophic neurites, ERK1/2 activity is lower recommending stage-specific ERK1/2 repression occurs after ERK1/2 activation (Webster et al. 2006).

Moreover, STZ inhibited Akt, preventing GSK-3β (Ser9) phosphorylation, which in turn increases GSK-3β (Tyr216), leading to the hippocampal apoptosis (Moosavi et al. 2014). Noteworthy, in STZ-induced diabetes in rodents, the inhibition of *p*-CREB activity and CREB-related expression of synapse protein in hippocampus as well as decreased BDNF activity were (Liu et al. 2020; Ripoli et al. 2020). Additionally, an in vitro study showed that oil of bergamot, the source of limettin, enhanced phosphorylation of GSK-3β at Ser9 by activation of Akt (Corasaniti et al. 2007). Also, Sustained activation of *p*-ERK1/2 leads to neuronal apoptosis via reducing *p*-GSK-3β (Ser9) expression, this was reversed by PD98059 and limettin treatment that downregulated *p*-ERK1/2 activation, hence increasing *p*-GSK-3β (Ser9) expression which displays beneficial effect against Aβ, leading to neuroprotection (Chuang et al. 2011). Consequentially, upregulated *p*-CREB (Ser133), had an impact on memory and learning (Brightwell et al. 2005), promoted BDNF production which has a neuroprotective, neuronal cell survival, and anti-inflammatory functions to suppress neuroinflammation and tau phosphorylation as seen herein. These were in comply with those studies that showed the influence of lithium, as GSK-3β inhibitor, on neuroblastoma SH-SY5Y human cells (Grimes and Jope 2001; Mai et al. 2002) as well as its effect in vivo and invitro in models of ischemic stroke induced excitotoxicity (Chuang et al. 2011). These studies dicuss how lithium enhance phosphorylation of GSK-3β at Ser9, thus increasing both BDNF and CREB. This goes along with an in vitro study used the cultured neonatal ventricular myocytes in rats in ischemia/reperfusion model showed that activation of *p*-ERK1/2 for long time, *p*-GSK-3β (Ser9) is reduced, while GSK-3β phosphorylation/translocation of Tyr216 increased, leading to cardiac cell apoptosis (Lin et al. 2011). Altogether with another study used insulin treatment in a cloned rat pheochromocytoma cell lines showed that increase in the *p-*GSK-3β (Ser9) levels agreed roughly with the repression in *p-*GSK-3β (Tyr216) because insulin activate Akt, an inhibitor for ERK1/2 as well as GSK-3β (Tyr216) (Krishnankutty et al. 2017).

Consequentially, upregulated immunohistochemical *p*-tau expression and increased Aβ production, thus cognitive decline was noted in SAD group. On the other hand, PD98059 relieved the aforementioned alterations. These effects were in comply with this in vivo study that used ICV-Aβ in brain to induce AD and how U0126, as ERK1/2 inhibitor, protected against Aβ toxicity and tau deposition(Ashabi et al. 2012).

Regarding neuroinflammation, STZ model group showed raised NF-κB, IL-6, and BACE-1 to add explanation of cognitive impairment occurred in this study. The presence of these inflammatory cytokines is a key characteristic of Alzheimer’s disease as they are one of important causes of memory impairment (Morales et al. 2014). This could be attributed through BACE-1 activation, resulting in amyloid plaque development that promotes the pro-inflammatory reaction and creates a viscous cycle as seen in this study and documented before (Qiao et al. 2021). Administration of PD98059 and limitten to the ICV-STZ group attenuated these neuroinflammation and Aβ deposition via reducing NF-κB, IL-6, and BACE-1contents. This goes align with the restriction impact of PD98059 on IL-6, NF-κB in an atopic dermatitis model (Yu et al. 2021), beside other studies documented the neuroprotective effect of PD98059 against BACE-1 expression in human SH-SY5Y neuroblastoma (Harrison et al. 2007) as well as Aβ precipitation (Rapoport and Ferreira 2000) in models of AD. Concerning STZ + limettin group, it combats against neuroinflammation and Aβ 40–42 aggregation to go with previous report (Kowalczyk et al. 2022b). Another in vitro study of chronic colitis model showed that earlier administration of citropten/ limettin reduced NF-κB and MAPK signaling pathway occurring in epithelium of intestine and triggered T cells (Lee et al. 2022b), in addition to reducing IL-6 in vascular inflammation model (Lee et al. 2022b). Also limettin and other coumarins like scoparone and umbelliferon have been reported to have antioxidant and anti-inflammatory activity showing neuroprotection (Kostova et al. 2012; Seong et al. 2019; Kowalczyk et al. 2022a; Lee et al. 2022a).

Likewise, in the current study, in addition to the biochemical changes, there are also histopathological and immunohistochemical changes as seen in ICV-STZ injected group that showed marked decrease in intact neuron cells and increased *p*-tau deposition in align with several studies (Abdallah et al. 2021; Sirwi et al. 2021). Contrary STZ + PD98059 and STZ + limettin groups improved these changes. This neuroprotection is corroborated by additional research reported the beneficial contribution of U0126 inhibitory effect on MAPK/ERK kinase in oxidative stress model using mouse and rat neuronal cell line (Satoh et al. 2000). Noteworthy, PD98059 shielded the mitochondrial system in brain’s cortex in a rat model of cardiac arrest (Zheng et al. 2020). Additionally, the potential of natural and synthetic coumarins on the CNS was documented *via* inhibiting cholinesterases and monoamine oxidase as well as microglial activation (Skalicka-Woźniak et al. 2016). Another study has shown that CA1 region of hippocampal slices in the male rats treated with 3–5 mg/kg dose of coumarin had a decline in nervous cells necrosis significantly using Nissle staining (Nasrin et al. 2021). Additionally, auraptene 25 mg/kg/day, an ingredient of many citrus fruits related to coumarines, had improved neuronal loss in hippocampus, seen by Nissle staining, in in vivo study of global ischemia mice model (Okuyama et al. 2013).

Building on earlier research, this study suggested a protective function of PD98059 and similarly limettin against SAD by targeting neuroinflammation caused by ERK1/2 as well as GSK-3β/ CREB/ BDNF pathway which contributes to AD etiology and progression.

## Conclusion

PD98059 and limettin ameliorated neuroinflammation and restored cell viability associated with improved histopathological and behavioral alterations as well as restoring AD hallmarks namely, Aβ and *p*-tau observed in SAD-induced in mice. These effects could be mediated through suppression of persistent activation of *p*-ERK1/2 which resulted in increasing *p*-GSK-3β (Ser9), leading to GSK-3β inhibition causing enhancement of CREB then BDNF expressions. Hence, based on these results, PD98059 and limettin may carry hope for protecting against SAD which may be through targeting p-ERK1/2/p-GSK-3β/p-CREB/BDNF pathway. Noteworthy, further studies using blocking/ inhibitory molecules to confirm neuroprotective effects through this pathway are encouraged. Moreover, further investigations are recommended to evaluate limettin activity in human subjects and its applicability as a possible treatment for SAD besides, evaluating the potential synergistic effects of PD98059 and limettin. Additionally, elaborating the conflict regarding ERK role which may vary according to the animal used, experiment duration and/or model, and cell types is suggested.

## Electronic Supplementary Material

Below is the link to the electronic supplementary material.

Supplementary Material 1

Supplementary Material 2

## Data Availability

Data is available upon request.

## Abbreviations

Aβ: Amyloid Beta
AChE: Acetylcholinesterase
AD: Alzheimer’s Disease
Akt: Protein Kinase B
ANOVA: Analysis of Variance
BACE-1: β-site Amyloid Precursor Protein Cleaving Enzyme 1
BDNF: Brain Derived Neurotrophic Factor
CREB: cAMP-Response Element Binding Protein
DMSO: Dimethyl Sulfoxide
ELISA: Enzyme-Linked Immunosorbent Assay
ERK: Extracellular Regulated Kinase
GSK: Glycogen Synthase Kinase
HRP: Horseradish Peroxidase
ICV: Intracerebroventricular
IL: Interleukin
i.p: Intraperitoneally
LTP: Long Term Potentiation
MAPK: Mitogen Activated Protein Kinase
MEL: Mean Escape Latency
MWM: Morris Water Maze
NF-κB: Nuclear Factor-Kappa B
*p*: Phosphorylated
PVDF: Polyvinylidene Fluoride
SAD: Sporadic Alzheimer’s Disease
SAP: Spontaneous Alteration Percentage
SDS-PAGE: Sodium Dodecyl Sulphate-Polyacrylamide Gel Electrophoresis
Ser: Serine
STZ: Streptozotocin
TEST: Tris-Buffered Saline with Tween 20 Buffer
