# Supplementary figures and images for: Limettin and PD98059 Mitigated Alzheimer’s Disease Like Pathology Induced by Streptozotocin in Mouse Model: Role of p-ERK1/2/p-GSK-3β/p-CREB/BDNF Pathway
Source: J Neuroimmune Pharmacol. 2025 May 17;20(1):55. doi: 10.1007/s11481-025-10211-8 (PMC12085375; doi:10.1007/s11481-025-10211-8)

**p-CREB (Ser133)**


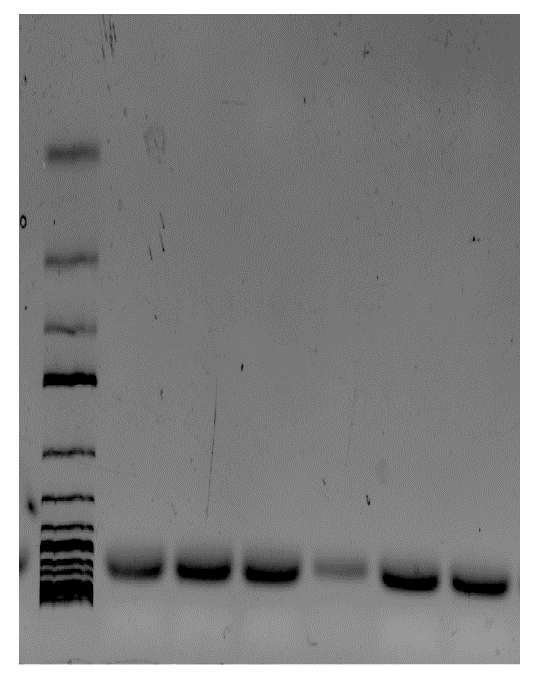

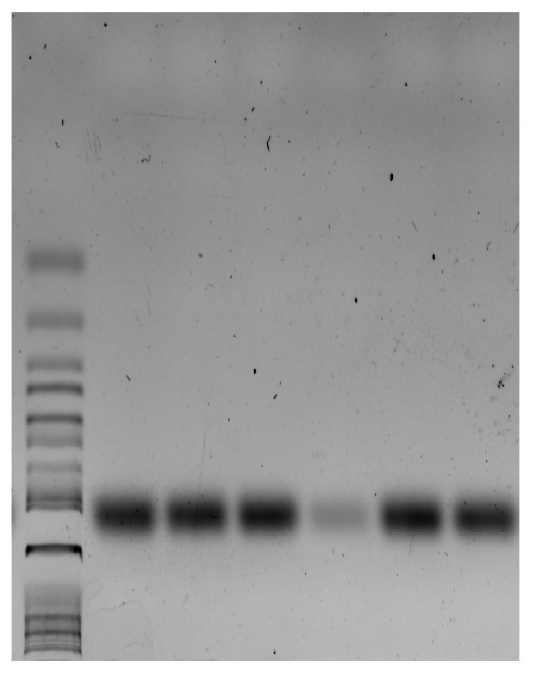

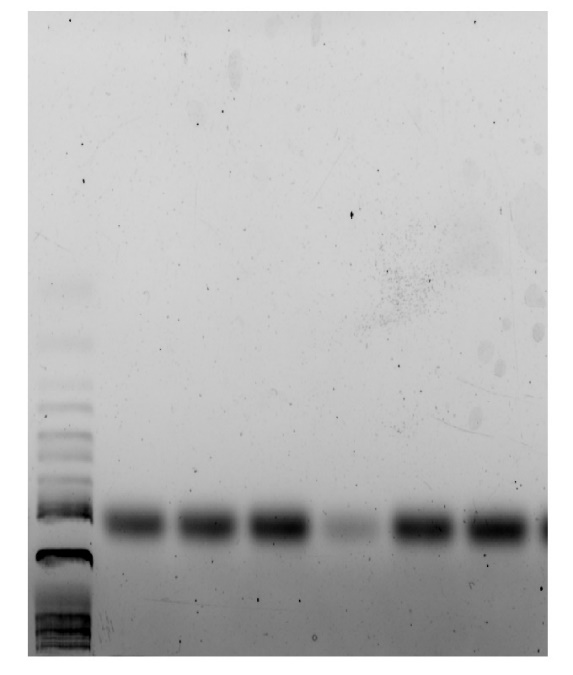


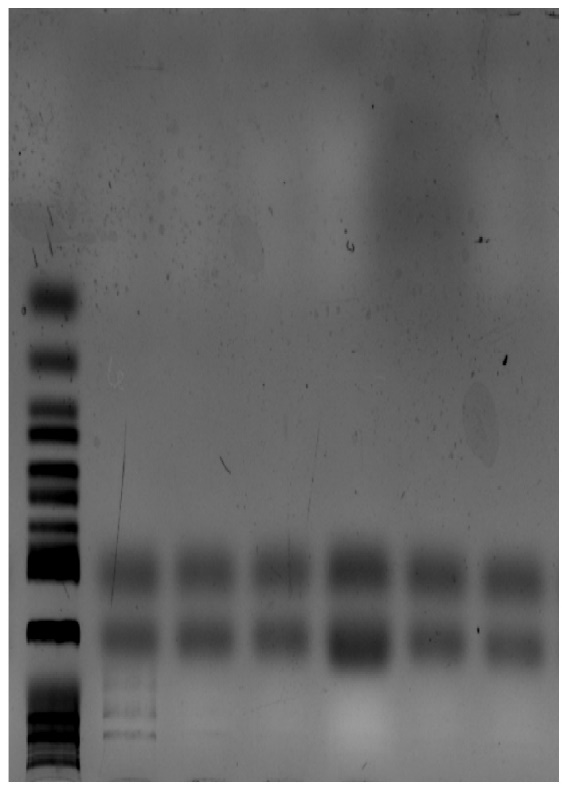
**p- ERK1/2**


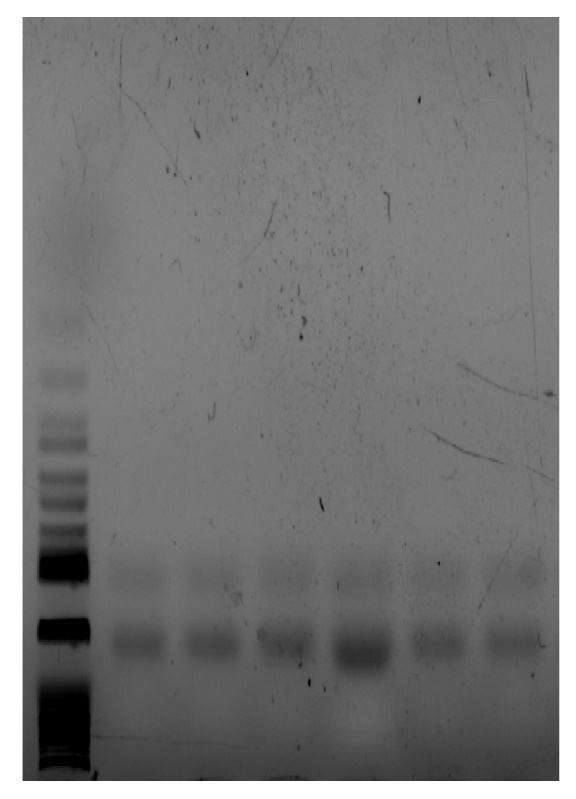

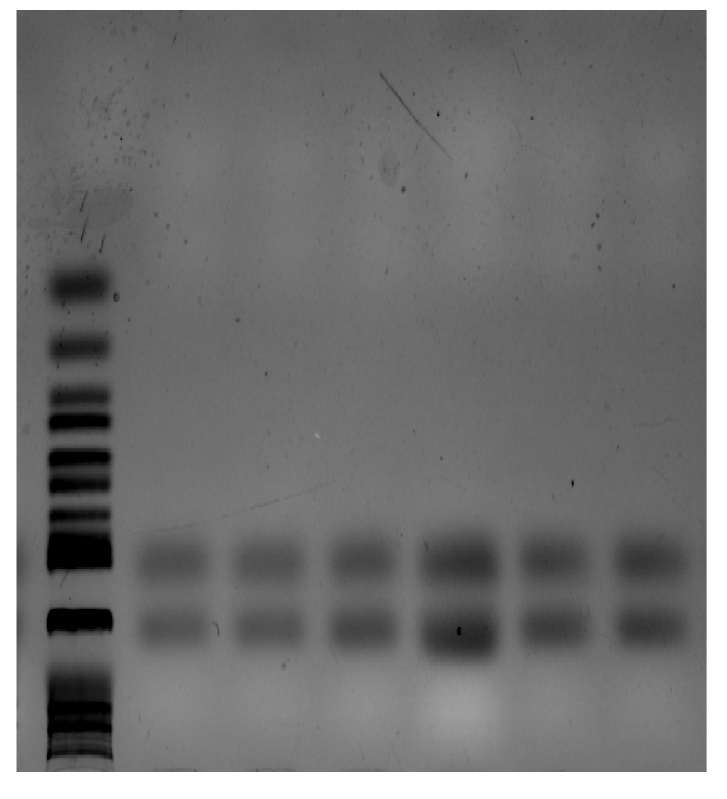


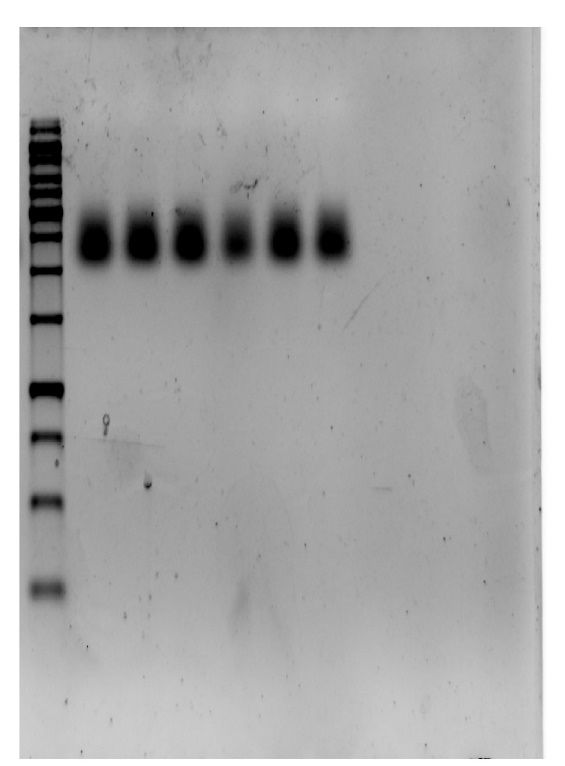

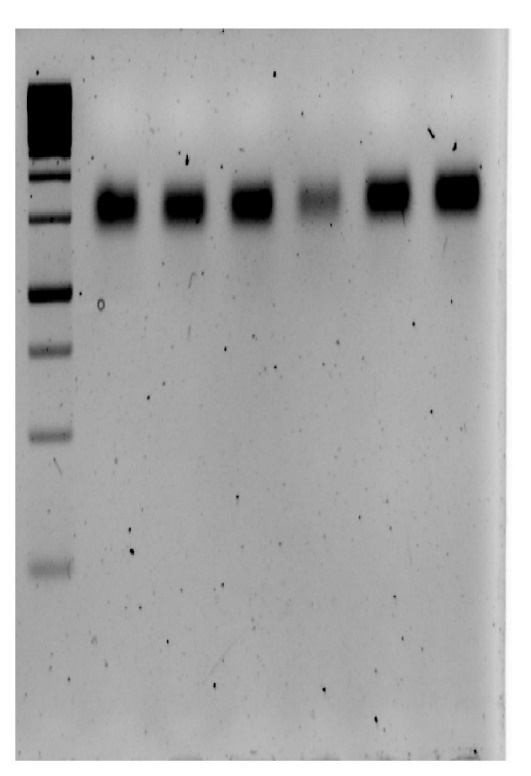

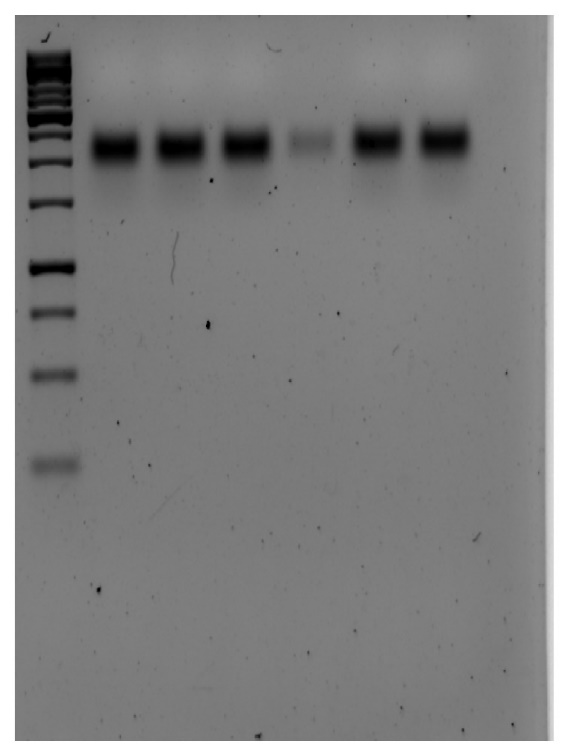
**p- GSK-3β (Ser9)**

Supplement: Supplementary file 1 — Supplementary Material 1 [file 11481_2025_10211_MOESM1_ESM.docx]
