# Supplementary material for: Limettin and PD98059 Mitigated Alzheimer’s Disease Like Pathology Induced by Streptozotocin in Mouse Model: Role of p-ERK1/2/p-GSK-3β/p-CREB/BDNF Pathway
Source: J Neuroimmune Pharmacol. 2025 May 17;20(1):55. doi: 10.1007/s11481-025-10211-8 (PMC12085375; doi:10.1007/s11481-025-10211-8)
Supplement: Supplementary file 2 — Supplementary Material 2 [file 11481_2025_10211_MOESM2_ESM.docx]

**Pilot Study**

For limettin, dose was selected according to a pilot study (supplementary data). In brief, according to (Lee, Kim and Jeong, 2022), authors used up to 40 mg/kg of limettin in DSS-Induced Colitis Model in mice, which showed beneficial effect, and toxicity wasn’t reported. Therefore, in our pilot study, we tested the effect of 2 doses (15, 30) mg/kg. The pilot study was designed as follows: Mice were divided into 4 groups (n=6/each); Control, STZ, STZ+limettin 15, and STZ+limettin 30, according to Y-maze and histopathological results, both doses showed similar effects, thus the dose 15mg/kg was selected.
